# A mixed methods, longitudinal study: characterizing the differences in engagement and perceived learning of medical students in online and in-person team-based learning classes

**DOI:** 10.12688/mep.19535.2

**Published:** 2024-02-12

**Authors:** Irene Cheng Jie Lee, Peiyan Wong

**Affiliations:** 1 MD Programme, Duke-NUS Medical School, Singapore, 169857, Singapore; 2 Department of Pharmacology, National University of Singapore, Singapore, Singapore

**Keywords:** TBL, SDT, motivation, cognitive strategies, community of practice

## Abstract

**Background:**

The rapid transition to online delivery of medical curriculum has facilitated the continuation of medical education during the COVID-19 pandemic. Whilst active learning approaches, including Team-Based Learning (TBL), are generally more supportive of the learner’s needs during such transition, it remains elusive how different learning environments affect a learner’s motivation, engagement, and perceived learning over a prolonged period. We leveraged on Self-Determination Theory (SDT) and key learners’ characteristics to explore the levels of student’s engagement and perceived learning in two TBL learning environments, online and in-person, over an extended period. We hypothesize that students’ self-reported perceptions of engagement and learning will be lower in online compared to in-person TBL classes.

**Methods:**

This is a mixed methods study with 49 preclinical graduate medical students completing the same questionnaire twice for each learning environment, online TBL and in-person TBL, over an eight-month period. Quantitative data were collected on learners’ characteristics, basic psychological needs satisfaction, motivation, student’s engagement and perceived learning. The final questionnaire also explored participants’ perception on which learning environment better supported their learning.

**Results:**

We found that autonomy support, perceived competence and needs satisfaction, and perceived learning were higher in-person than online. Additionally, most learners felt that in-person TBL was better for learning, as the concepts of learning space and the community of practice were mediated by being in-person.

**Conclusions:**

TBL, being an active instructional method, can maintain students’ engagement because it supports many aspects of SDT constructs and perceived learning. However, online TBL is unable to fully support the students’ needs and perceived learning. Hence, we strongly advocate for any in-person opportunities to be included in a course, as in-person classes best supports students’ engagement and perceived learning.

## Introduction

Enhanced social distancing measures due to the COVID-19 pandemic had transformed the delivery of education, from onsite physical lessons to online settings ^1– 3^. Due to the looming threat of infectious variants and the rapidly changing pandemic situation, many educational institutions implemented either online learning or a hybrid arrangement of blended in-person and online learning for a prolonged period of time ^4^. The increasing number of reports on the lessons learnt and efforts in the online transition ^1, 5^, particularly in terms of learners’ perception of the online learning environment ^3^, had enabled the swift implementation of online learning. However, it remains unclear whether being in an online learning environment over an extended period changes a learner’s motivation and in turn, the level of engagement and the perception of learning. Hence, gaining a better understanding of the effects of learning environments on students would help to identify key elements needed to support learners in a prolonged online learning environment, and inform future integration of online learning into the existing medical curriculum.

At Duke-NUS Medical School, Singapore, we adopt Team-Based Learning (TBL) as our main instructional method in the preclinical curriculum. We have previously shown that a week-long, online learning experience did not affect students’ perception of their classes ^2^. However, the online learning classes in that study did not encompass all aspects of a typical TBL session, as no video-conferencing was used, therefore there was no active discussion between students and faculty. Furthermore, there is a lack of research on the effect of online versus in-person TBL on students’ engagement and perceived learning. Hence, in this study, we used the self-determination motivational theory framework, together with key learner’s characteristics, to identify changes to the factors underlying learners’ engagement and perceived learning when taking classes in two different learning environments, over an extended length of time.

### Theoretical framework

According to Self-Determination Theory (SDT) motivational theory, motivation is a continuum, ranging from amotivation, a lack of motivation state, to extrinsic motivation which is propelled by an external factor or reward, and then to intrinsic motivation, a self-generated state ^6^. Intrinsic motivation, which is the pursuit of an activity for one’s personal interest, is shown to associate with better learning outcomes and well-being ^7– 10^. Such motivation fosters engagement on any task. SDT further differentiates the types of extrinsic motivation based on the level of autonomy ^6, 11^. External regulation, the least autonomous one, is outside of personal control and driven by external reward or avoidance of punishment, whereas identified regulation, the most autonomous one, is associated with valuing an activity. Both intrinsic motivation and identified regulation are the autonomous types of self-determination for a learner.

Importantly, SDT hypothesizes that learner’s motivation on any given task can change from extrinsic to intrinsic, depending on the satisfaction of the learner’s three basic psychological needs: autonomy, competence, and relatedness. Autonomy refers to acting with a sense of volition; competence is self-perceived efficacious in a learning environment; relatedness is the feeling of being connected and belonging to other or one’s community. It was further demonstrated that students are more able to stay motivated and engaged in a learning task when instructional method fulfills these psychological needs ^12^. Furthermore, when in-person, the TBL instructional method was better, than didactic instructional method, at fostering intrinsic motivation, perceived competence, autonomy and overall needs satisfaction ^13^. Using the SDT framework, it helps us to understand why learners stay engaged and develop greater sense of perceived learning in a TBL environment.

While SDT is important in shaping the fundamental aspects of engagement and learning, research has shown that there are other factors, such as learners’ characteristics, that interact with the SDT framework and motivation via their correlations with the basic psychological needs satisfaction. These key characteristics include curiosity, resilience, and growth mindset ^14– 20^. Pintrich (1990) also suggested that motivated students were cognitively engaged and more likely to apply meta-cognitive learning strategies, becoming self-regulated learners ^21^. Hence, we included the use of cognitive strategies and self-regulation in our theoretical framework ^21^.

**Figure 1.**
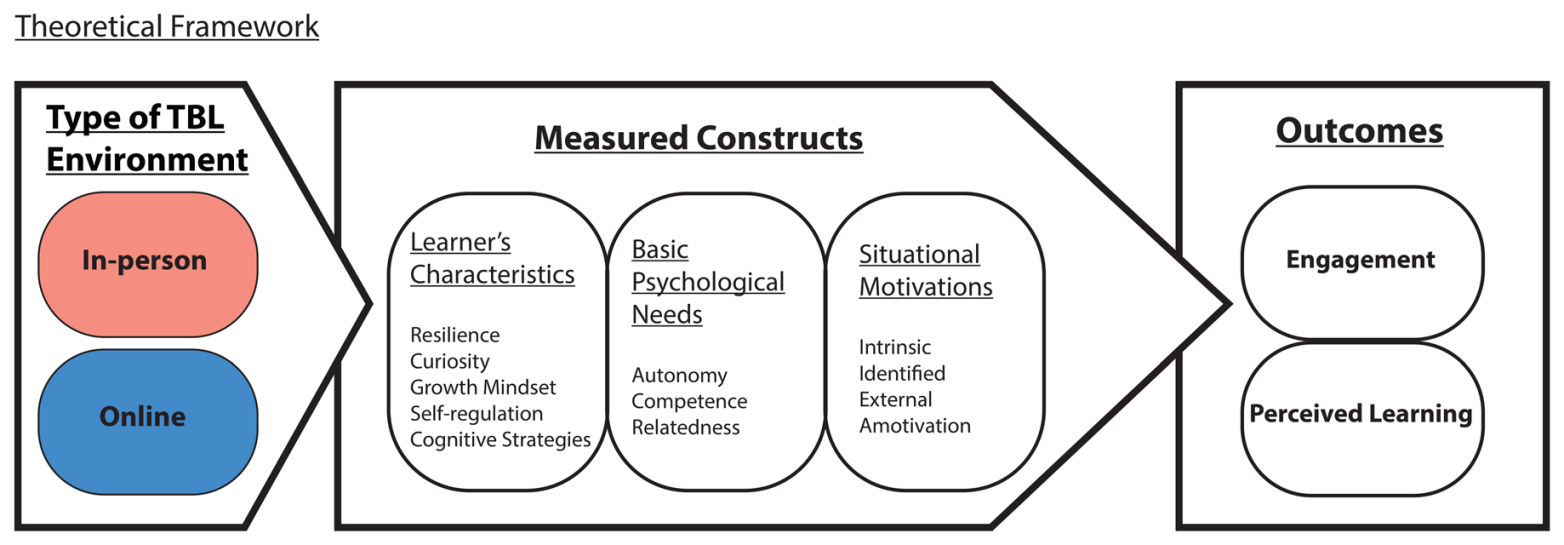
Theoretical framework to elucidate student’s engagement and perceived learning (measured outcomes) via the measured SDT and learner’s characteristics constructs in the in-person and online TBL learning environments.

Given the dynamic relationship between the learning environment and various learners’ characteristics, and the satisfaction of the basic psychological needs for motivation, we seek to elucidate how these factors influence learners’ engagement and perceived learning ( Figure 1). We posit that the reduced or lack of social interactions of the students, with their team members and faculty, in an online TBL learning environment might lead to reduced satisfaction of basic psychological needs compared to an in-person TBL learning environment. Hence, students’ engagement and perceived learning in online TBL will be adversely affected.

Specifically, we aim to characterise the differences in learners’ characteristics (such as resilience, curiosity, growth mindset, use of cognitive strategies and self-regulation), basic psychological needs satisfaction (autonomy, competence and relatedness), situational motivation (intrinsic, identified, external and amotivation), student’s engagement and perceived learning between the two TBL learning environments, online and in-person, over an extended period of time of about four months in each environment. Understanding such differences in learning environment will inform the academic support system on how best to engage learners in their learning environment. Besides, key elements identified in this relationship will also facilitate integration of online TBL learning into existing medical curriculum in this post-COVID-19 era.

## Methods

### Participants

We recruited, via e-mail, 49 participants from a pool of 72 graduate medical students (comprised of 45 females and 27 males), who were in the first-year of their studies at Duke-NUS Medical School. There were 30 females and 19 males who participated in our study. Their age distributions were as follows: 24 participants were between the ages of 21–25 and 25 participants were over the age of 26.

### Context of the learning environment: Preclinical Curriculum

Our preclinical curriculum integrates the basic science content into a one-year programme, Foundations of Patient Care (FPC), which is covered in two parts: (1) FPC Part 1 adopts an integrated, systems-based approach to study the human body’s structure and function, (2) Part 2 then progresses to a systems-cutting approach, focusing on disease. This developmental framework is designed to first establish an understanding of the "normal" body before delving into the "abnormal," while also introducing essential clinical reasoning skills. To deliver this curriculum, we utilize a range of teaching and learning methods with TBL as one of the main strategies. The curriculum assesses students with summative high-stake knowledge and clinical performance assessments.

### Recruitment procedure

This mixed-methods, quasi-design study used survey methodology and was exempted by the National University of Singapore Institutional Review Board (NUS-IRB-2020-346). An administrative staff member with no grading responsibilities emailed all first-year graduate medical students with links to the questionnaire, consisting of demographic questions and validated survey instruments measuring the various constructs (detailed below). Participants indicated their informed consent electronically before completing the questionnaire. 

### In-person and online TBL instructional methods

**Figure 2.**
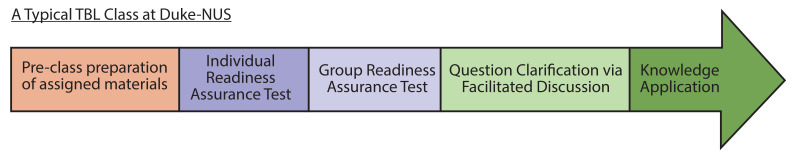
An infographic showing the sequence of events that happens for a TBL class at Duke-NUS. Students undergo these TBL phases for all classes in the first year of medical school at Duke-NUS, regardless of whether the classes were held online or in-person. The way that pre-class preparation materials were assigned, and the TBL-supported software used for the assurance tests were the same in both learning environments. Please see Lee *et al.*, 2022 for a review comparing the resources required for each learning environment ^4^.

At Duke-NUS, TBL is the main instructional method ^22^ for first year classes, regardless of whether they are online or in-person ( Figure 2). In brief, for a typical TBL class at Duke-NUS, students study the assigned learning material prior to classes. During class, students take the readiness assurance tests individually and then as a team. After which, there is a clarification phase for students to clear up their remaining doubts, and their queries would be addressed as a class during an instructor-facilitated discussion. Both the online and in-person TBL classes consist of all the phases of a typical TBL class, except that in an online TBL class, a video-conferencing software, Zoom, would be used ^1^. Students would be put in breakout rooms when they are doing the team readiness assurance tests and working through the clarification questions. The instructor-facilitated discussions would follow on Zoom. When in-person, the whole team would be in the classroom and students are able to carry out group discussions with their teammates face to face. The administrative staff and facilitating instructors would be present in the classroom.

### Experimental design

**Figure 3.**
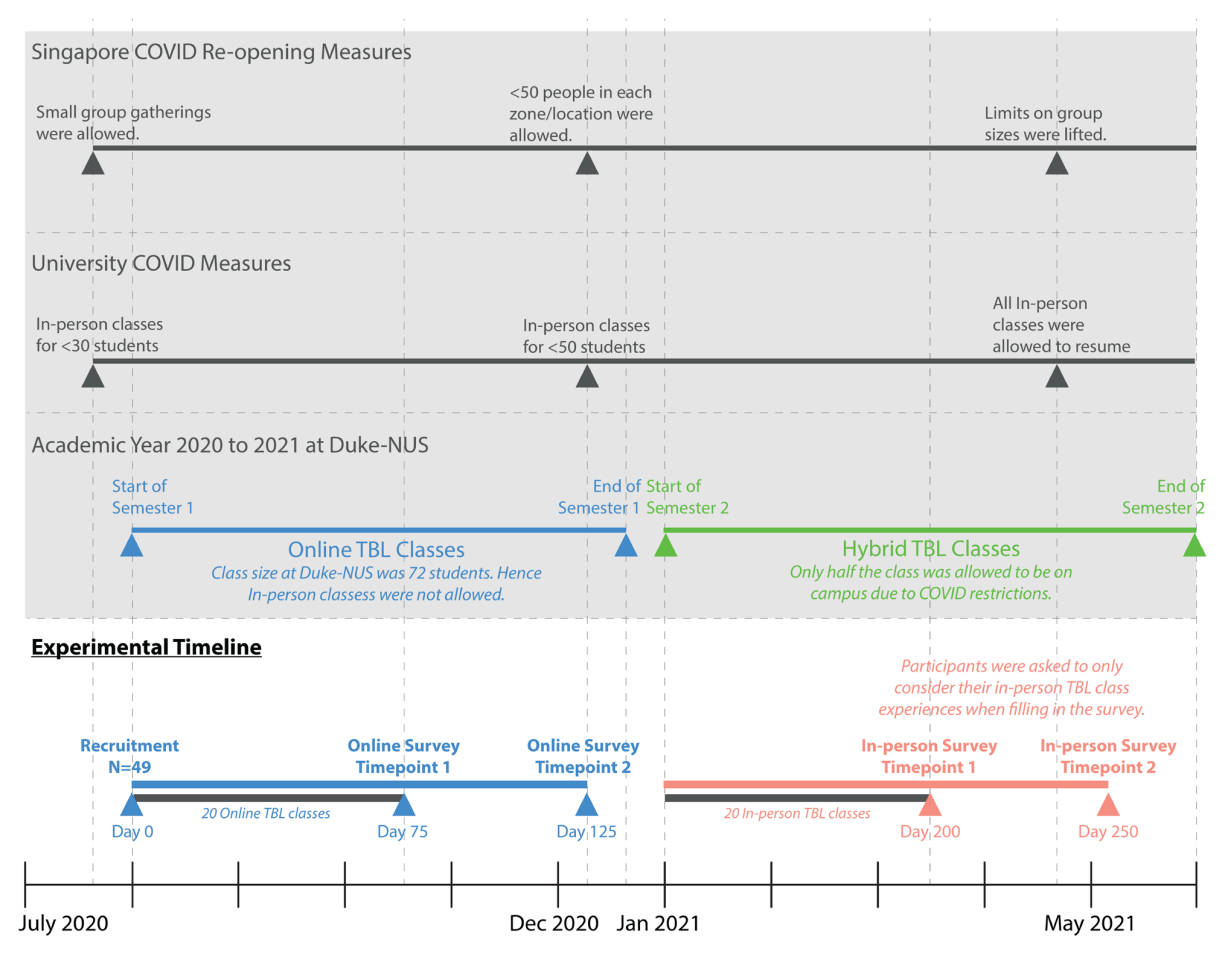
Timeline of the experiment showing when the four surveys, two for each learning environment, were administered. The social distancing requirements by the Singapore government and the university were included to provide context for why and when classes were online and in-person, in a hybrid format. All first-year classes were carried out using the TBL format. This cohort of students started TBL classes online in August of 2020 due to the high number of cases in Singapore. In January 2021, synchronous hybrid TBL classes were implemented for this cohort, when cases were lower and social distancing measures were relaxed. Students followed a roster for when they were supposed to attend classes in-person (see Figure 4).

In our study, all 49 participants completed a total of four surveys, interspersed throughout the academic year, with two surveys administered for each learning environment, online and in-person ( Figure 3). The first survey for each learning environment was administered after twenty TBL classes in that learning environment (day 75 for online and day 200 for in-person). This was to allow sufficient time for participants to get to know their teammates and immerse in the TBL instructional method ^23^. The second survey for each learning environment was administered fifty days later, to assess for any changes after a longer exposure in each learning environment. During the fourth and final survey, we included additional questions that explored the participants’ perception of their overall learning experiences in both online and in-person TBL, by asking them to indicate which learning environment they thought they learnt better in and why.

**Figure 4.**
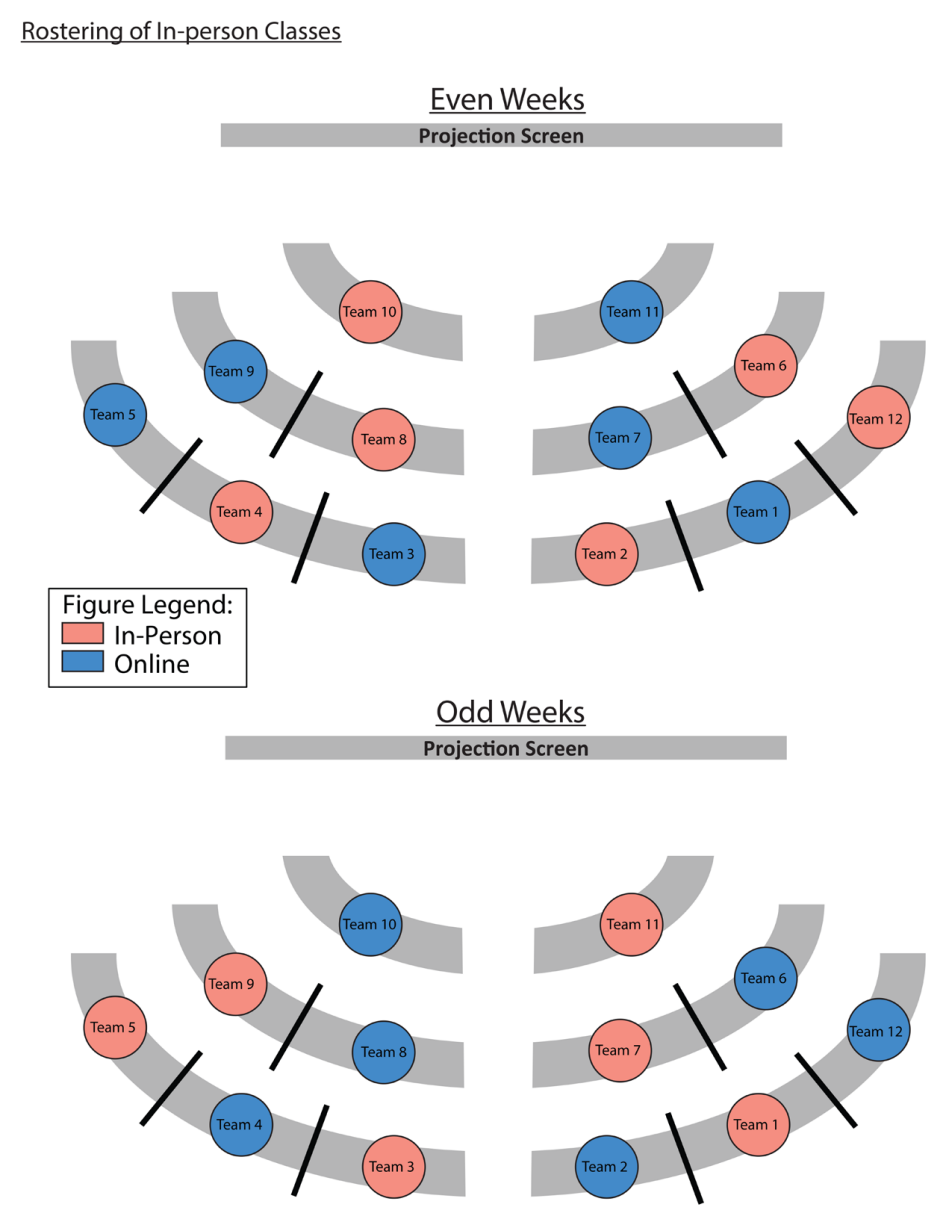
An example of how the teams were rostered for in-person and online classes. Due to the government-imposed, social distancing requirements, the classroom at Duke-NUS was only able to accommodate half of the first-year cohort at any given time. Hence, we adopted a synchronous, hybrid TBL teaching, and a roster, for teams to attend in-person or online classes, was implemented to ensure that every student had the same number of in-person and online sessions. All students underwent live classes, held at the same time, regardless of whether they rostered to be in-person or online. If the team was rostered to have in-person classes (blue circles), students were expected to be in the classroom, unless they were under quarantine. The teams rostered to have online classes (pink circles), would take part in the lessons from home.

All first-year classes were carried out using the TBL format. This cohort started TBL classes online in August of 2020 due to the high number of cases in Singapore. The first survey for online TBL classes was administered 75 days after their first online TBL class, and the second survey followed 50 days later. In December 2020, synchronous hybrid TBL classes were implemented for this cohort, when cases were lower and social distancing measures were relaxed. Students followed a roster for when they were supposed to attend classes in-person (see Figure 4). The first survey for in-person TBL classes was administered 75 days after their first hybrid TBL class, and the second survey followed 50 days later.

During this study period, the participants started their Year 1, semester 1, in the online TBL environment, due to the heightened social distancing measures during the COVID-19 pandemic. In semester 2, as the infection clusters came under control, social distancing measures were relaxed sufficiently for half the cohort to attend lessons in-person, with the other half attending synchronously on Zoom. Details of how the hybrid TBL class was carried out was published elsewhere ^4^. To ensure equal exposure for both halves of the cohort to in-person TBL classes, students were rostered, by teams, to come to school on alternate weeks ( Figure 4). Due to this hybrid class arrangement, participants were reminded to only take into consideration their experiences for the in-person sessions when completing the surveys (see *Extended data* ^24^) for the in-person learning environment.

### Scales used

At each sampling timepoint, the questionnaire measured all learner’s characteristics (resilience, curiosity, growth mindset, use of cognitive strategy and self-regulation), basic psychological needs constructs (autonomy, perceived competence, basic needs satisfaction), situational motivation constructs, and engagement and perceived learning (see *Extended data* ^24, 25^). All these were validated survey instruments.

*Learner’s characteristics*: Students’ resilience was measured using six-item Brief Resilience Scale ^26^. Mindset of students was determined using three-item Growth Mindset Scale ^27, 28^. Curiosity of students was measured using 5D Curiosity Scale that focuses on five-item joyous exploration ^19^. Self-regulated learning of students was measured using Motivated Strategies for Learning Questionnaire on the subscale of twelve-item cognitive use strategy and nine-item self-regulation ^21^.

*Basic psychological needs and situational motivation constructs*: Students’ perception of the instructor’s autonomy support was measured using six-item Learning Climate Questionnaire ^13, 29, 30^. Students’ perceived competence was measured using four-item Perceived Competence Scale ^30^. Students’ needs satisfaction in general was measured using Basic Psychological Needs Scale ^31, 32^ which is made up of three subscales: autonomy; competence; relatedness. The three subscales were combined to measure a general needs satisfaction scale. To measure students’ situation motivation during the learning environment, we used a sixteen-item Situational Motivation Scale which determines the level of intrinsic motivation, identified regulation, external regulation and amotivation ^33^.

*Outcome measurements of perception of engagement and learning:* 22-item Multidimensional Engagement scale was used to measure four aspects of students’ engagement including agentic engagement, behavioural engagement, emotional engagement and cognitive engagement ^34^. Nine-item CAP Perceived Learning Scale was used to measure perceived cognitive, affective and psychomotor learning gain ^35^.

### Quantitative data analysis

The R statistical program (R Foundation for Statistical Computing, Vienna, Austria), version 3.6.2, was used for data analyses. Data were expressed as means ±SEM and p<0.05 was considered statistically significant. As all participants completed every administered survey, two for each learning environment, we conducted a within subjects ANOVA with repeated measures to examine the differences in measured constructs in the in-person and online learning environment across the timepoints. The normality of residuals and homogeneity of variance were checked to ensure that there were no violations before the ANOVA was run. We also calculated the Cronbach’s Alpha to assess the internal consistency of each scale and used Cohen’s *d* to measure the effect sizes for mean differences ^36^. The R packages used to run the analyses were as follows: *OLS regression*, the Levene’s test from the *car* statistical package, *lmerTest* statistical package, *anova()* function from the *stats* package, *cohen.d()* function from the *effsize* package and *Cronbach.alpha()* function from the *ltm* package ^37, 38^.

### Thematic analysis

We used thematic analysis to explore the reasons why students perceived a particular environment better for learning. We used an inductive, reflexive thematic analysis through the 6 phases for thematic analysis ^39^. The author familiarized with the data, taking casual observational notes about the content. This was followed by inductive question informed coding, at both sematic and latent levels. Themes were then constructed from these codes and their associated data, first, by establishing candidate themes, which were tested for their utility in telling the story of the data, then reviewing and finalizing themes. Two authors discussed the content of codes and candidate themes using thematic maps to make sense of them. We generated two themes from the data describing the reasons underlying the better learning in a particular environment.

## Results

### Reliability statistics

Internal reliability of survey instruments was compared to previous values reported in the literature. The obtained Cronbach’s alpha values for all the survey instruments were indicated in Table 1, together with Cronbach’s alpha values that have been reported in the literature. In short, the obtained reliability statistics values in our study were comparable to previously reported values.

**Table 1.** Reliability statistics of the survey instruments used in this study in comparison to previously reported values.

Measured Constructs	Cronbach’s α	Published Cronbach’s α	References
**Learner’s Characteristics**			
Resilience	0.86	0.8-0.91	26
Growth Mindset	0.94	0.94-0.98	27
Curiosity	0.88	0.87-0.9	19
Cognitive Strategy	0.74	0.76-0.83	41
Self-Regulation	0.73	0.74	21
**Basic Psychological Needs**			
Autonomy	0.87	0.75-0.93	13, 29
Needs Satisfaction	0.74	0.66-0.86	42, 43
Perceived Competence	0.81	0.8	30
**Situational Motivations**			
Intrinsic Motivation	0.66-0.89	0.58-0.93	13, 33
Identified Regulation			
External Regulation			
Amotivation			
**Outcomes**			
Engagement	0.76	0.78-0.94	34
Perceived Learning	0.64	0.79	35

### Effect of learning environment on variables of the learner’s characteristics and self-determination theory framework

All variables showed homogeneity of variance. The residuals of cognitive strategy, self-regulation, identified regulation, external regulation and perceived learning did not follow normal distribution due to the presence of outliers. The removal of these outliers, as identified by Cook’s Distance ^40^, showed that these observations did not have any influence on the statistical analysis model. As such, these observations remained in the analyses. Our ANOVA analysis showed that within each learning environment, in-person and online, there were no significant differences in each of the measured variables between the first and second timepoint ( Table 2). Since there were no significant differences between the early and late survey timepoints for each variable within each learning environment, they were averaged for subsequent analysis. There were also no detectable differences between male and female participants, and hence, the data were aggregated for subsequent analyses.

**Table 2.** Means, standard error of means for the constructs measured at the early and late timepoints in the in-person and online TBL learning environment.

Measured Constructs	In-Person 1 (Day 75)		In-Person 2 (Day 125)		Online 1 (Day 75)		Online 2 (Day 125)	
	Mean	SEM	Mean	SEM	Mean	SEM	Mean	SEM
**Learner’s Characteristics**								
Resilience	3.74	0.09	3.69	0.11	3.79	0.08	3.81	0.09
Growth Mindset	4.40	0.22	4.25	0.23	4.58	0.20	4.34	0.20
Curiosity	5.44	0.13	5.51	0.13	5.43	0.13	5.46	0.10
Cognitive Strategy	5.03	0.09	5.08	0.10	5.05	0.10	5.04	0.08
Self-Regulation	5.11	0.10	5.20	0.10	4.99	0.09	4.99	0.10
**Basic Psychological Needs**								
Autonomy	5.32	0.11	5.28	0.12	5.18	0.13	4.89	0.10
Needs Satisfaction	3.76	0.07	3.79	0.07	3.42	0.09	3.54	0.07
Perceived Competence	4.61	0.09	4.67	0.10	4.32	0.12	4.37	0.10
**Situational Motivations**								
Intrinsic Motivation	5.36	0.14	5.33	0.14	5.50	0.15	5.13	0.13
Identified Regulation	5.99	0.11	5.95	0.09	5.97	0.09	5.85	0.10
External Regulation	5.04	0.13	4.95	0.18	4.99	0.14	5.28	0.12
Amotivation	2.46	0.17	2.43	0.17	2.28	0.16	2.51	0.15
**Outcomes**								
Engagement	5.27	0.10	5.27	0.08	5.28	0.08	5.12	0.08
Perceived Learning	3.58	0.09	3.59	0.07	3.22	0.09	3.38	0.08

**Figure 5.**
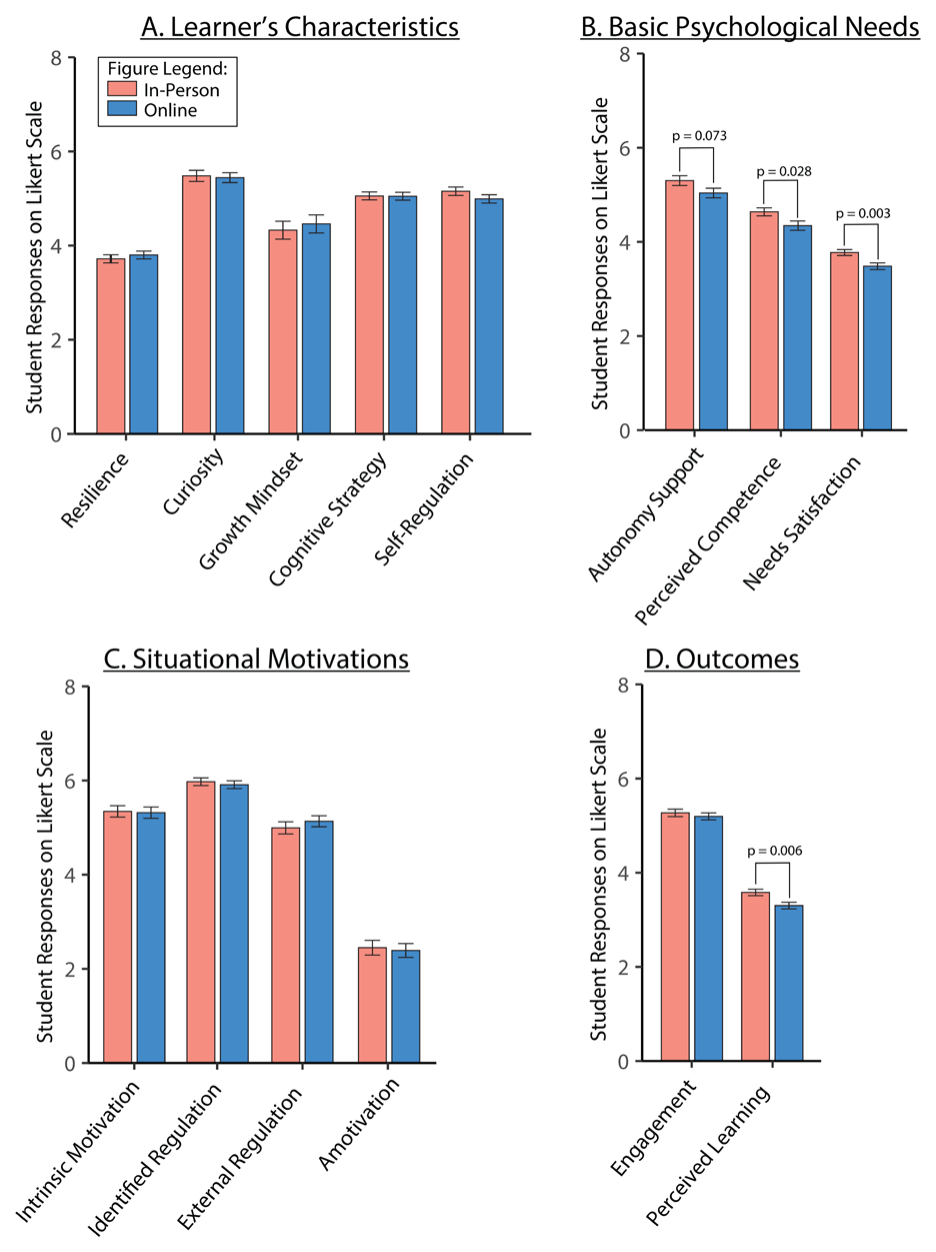
*Panels **A** to **D**.* Measured constructs for in-person (pink bar) and online (blue bar) TBL learning environment. Averaged scores are shown for learner’s characteristics constructs ( *Panel **A***), and basic psychological needs ( *Panel **B***), situational motivations ( *Panel **C***) and measured outcomes ( *Panel **D***). Effect sizes (Cohen’s d) for differences between in-person and online: autonomy support, *d* = 0.367; needs satisfaction, *d* = 0.620; perceived competence, *d* = 0.446; perceived learning, *d* = 0.569. Data is represented as means and standard error of mean.

We assessed the effect of learning environment on the five variables from the learner’s characteristics framework: resilience, growth mindset, curiosity, cognitive strategy, and self-regulation ( Table 3). The ANOVA analysis of averaged responses showed no differences between in-person and online TBL classes for learner’s characteristics ( Figure 5A).

**Table 3.** Means and standard error of means for learner’s characteristics, basic psychological needs and situational motivation constructs, and study outcomes (engagement and perceived learning) averaged across the two timepoints. *These measured variables showed significant differences between in-person and online learning environments (ps<0.05).

Measured Constructs	In-Person		Online	
	Mean	SEM	Mean	SEM
**Learner's Characteristics**				
Resilience	3.72	0.09	3.80	0.08
Growth Mindset	4.33	0.19	4.46	0.19
Curiosity	5.48	0.12	5.44	0.11
Cognitive Strategy	5.05	0.08	5.05	0.08
Self-Regulation	5.15	0.09	4.99	0.09
**Basic Psychological Needs**				
Autonomy	5.3	0.10	5.04	0.10
Needs Satisfaction *	3.77	0.06	3.48	0.07
Perceived Competence *	4.64	0.09	4.34	0.10
**Situational Motivations**				
Intrinsic Motivation	5.34	0.12	5.32	0.12
Identified Regulation	5.97	0.08	5.91	0.08
External Regulation	4.99	0.13	5.14	0.12
Amotivation	2.45	0.16	2.39	0.15
**Outcomes**				
Engagement	5.27	0.08	5.20	0.07
Perceived Learning *	3.58	0.07	3.30	0.07

We also assessed the effect of learning environment on the basic psychological needs and situational motivation variables ( Table 3, Figure 5B and 5C). Perceived competence and needs satisfaction showed significant main effects of learning environment ( Figure 5B; p = 0.028 and p = 0.003 respectively), with in-person being higher than online. Autonomy support showed increases with in-person compared to online, although this was not significant (p = 0.073). There were no significant changes detected in the situational motivation variables: intrinsic motivation, identified regulation, external regulation and amotivation ( Figure 5C).

Of our outcome measures, engagement and perceived learning, only perceived learning showed significant main effect of learning environment ( Table 3, Figure 5D; p=0.00584), with in-person being higher than online.

### Which learning environment did students feel that they learnt better in?

**Figure 6.**
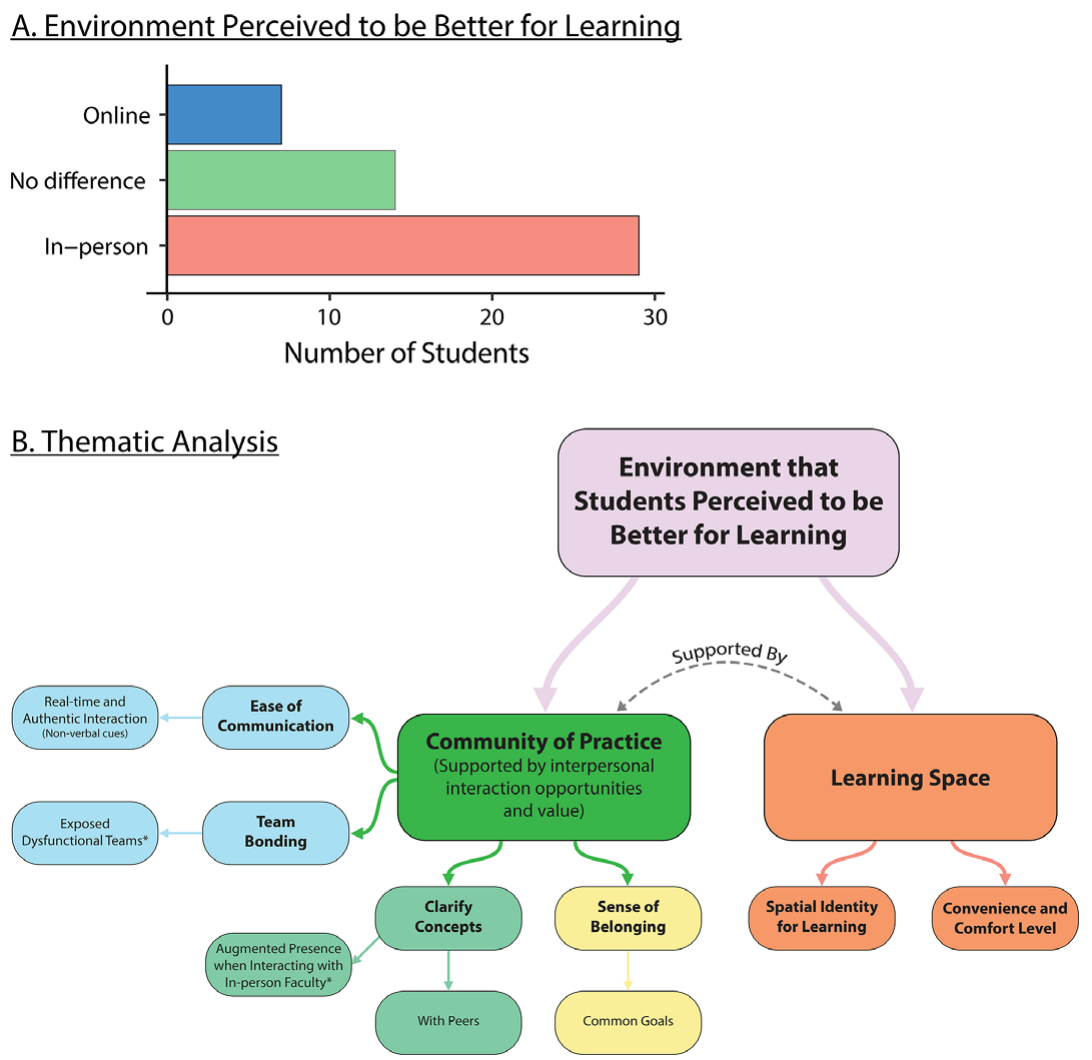
*Panel **A**.* Most of the participants perceived in-person classes to be better for learning than online classes. *Panel **B**.* A thematic analysis showed two main themes of why students perceived a particular environment to be better for learning. The community of practice supported by interpersonal interaction opportunities and the learning space played crucial roles in shaping the students’ perception of which learning environment was better for learning.

At the last survey timepoint, we asked students to reflect upon their class learning experience and choose which environment they thought they learnt better in. From the 49 responses, 29 picked in-person TBL, 14 did not have a preference, and 6 picked online TBL ( Figure 6A). We did not find any impact of age (21–25, >26) and gender differences on the preferred learning environment.

The comments reflected two themes that shaped a student’s perception of which learning environment was superior ( Figure 6B). Each theme cohered around a central organising concept that underpins the thematic explanation of the data: (1) Being in the community of practice, supported by the interpersonal interaction opportunity and its associated value, influences students preference of a learning environment in learning (2) How learning space directly impacts students’ ability to learn and focus.

The *main theme* is the ***community of practice, supported by the interpersonal interaction opportunity and its associated value*** that is offered in that learning environment.

A majority of students valued such community of practice as evident by the interpersonal interaction amongst peers which was facilitated during in-person TBL. Students perceived ease of communication, reflected by the authenticity of non-verbal cues and real-time feedback, to support team building and bonding during in-person TBL. For example, these are the comments by participants favouring in-person TBL:

*“Face-to-face TBL is more convenient and conducive way to work together with our team members and our classmates. It is easier to forge friendships and learn from one another.”*
*“Zoom has its limitations in team learning- it doesn't feel as "natural" and it is lacking in the nuances of non-verbal communication. I feel more engaged during face-to-face discussions, either with my team or with the faculty facilitator during teamlead sessions.”*

The ability to clarify concepts either with peers or faculty during in-person also shapes such perception of community of practice. Students favoured in-person TBL given the flexibility to clarify concepts with peers face-to-face. For instance,

*“…can ask for help from friends whenever we have questions or didn't catch what the lecturer said.”*
*“Discussion during GRA is more conducive in person where it is easier to express our thought process and interject each other”.*

However, such interaction with faculty remains limited as the faculty did not co-share the same physical learning environment with the students, given the prevailing social distancing measures on teaching venue. Thus, this was highlighted in students who found no difference between the two learning environments. Such sentiments were reflected by students who commented the following:

*“Both modalities feel rather the same to me with the main bulk of the teaching done by the professors and doctors over zoom and the location where I am receiving and learning such information does not really matter much to me”*.
*“I miss the moments when after class we can go up to the lecturer to ask questions freely + listening to the lecturer interacting with us answering our questions.”*

Additionally, another critical sub-theme was sense of belonging as students perceived having common goals of working and learning together being possible only with in-person TBL.

*“It is easier to concentrate when physically surrounded by classmates focusing on doing the same thing.”*
*“..because I like the atmosphere of doing the same thing achieving the same goal with a crowd of people.”*

While most students who supported in-person TBL due to the abundance of interpersonal interaction with peers, a handful of students preferred online TBL as a result of dysfunctional team dynamics presented over the initial phase of prolonged online learning environment. For examples, these two students perceived being disconnected from team members and not being engaged in an in-person TBL, suggestive of inherent challenges with team cohesion and dynamics.

*“Don't really feel connected to teammates… Better to focus at home instead”*
*“While discussing things over zoom, everyone is on the same page whereas in school, discussions will leave some members out”.*

The second theme, ***learning space***, also plays crucial role in shaping students’ preference of a learning environment better for learning.

Students perceived the importance of spatial and physical space for learning, by which campus provides that unique identity for students to stay focused as shared by the following comments:

*“…less distracted in person because the environment is encouraging you to pay attention to the most salient thing around you which is the lecture (on the projector)”*.
*“The school's environment is more conducive for learning, and I get into a state where I know "it's time to study".*

On this note, such spatial identity for learning, in part, supports the main theme via providing the opportunity for community of practice.

*“Easier to keep engaged/pay attention during physical lessons, being in a physical study space surrounded by peers and faculty is much more conducive for learning and retention.”*

Similarly, some students experienced more distractions at home, negatively impacting their learning and preference as shown below:

*“At home unengaged, mixed environment cues from bedroom, distractions of home and family,”*
*“My home environment is not optimized for online TBL, and there are too many distractions (construction outside my house, parents also working on call, internet instability, etc) for online TBL to be effective”*

On the other hand, students, who favoured online TBL, enjoyed the direct benefits of home learning environment including convenience and comfort. Students valued more time studying at home as it saved from travelling to campus.

*“Less time travelling = more time studying”*
*“…home-based learning is less distracting, allows you to study more efficiently during breaks, and allows me to use my own work desk with laptop stand, external keyboard, mouse and footstool etc. in comfort so I can study more effectively.”*

While these two themes shape and influence how students favoured a particular learning environment, it also emphasized the critical element of satisfying the needs of being part of community of practice supported via interpersonal interaction.

## Discussion

Previous studies have shown that instructional methods and environment influence self-determination theory constructs, which in turn affect engagement and perceived learning ^13, 44^. Ours is the first longitudinal study we know of that looked at whether the learner’s characteristics and SDT constructs could be impacted by the type of TBL learning environment over time.

### Although self-determination theory constructs were stable across time in an online learning environment, some were lower than in-person

In this study, we showed that the learner’s characteristics and self-determination theory constructs did not change across time in each learning environment ( Table 2). More importantly, these constructs remained stable across time, within the online learning environment, irrespective of the subject matter being learnt, as the participants were undergoing different courses at each survey timepoint. Our observation of the stable behaviours in the online learning environment contrasts with previous studies, which showed that online courses that used didactic instructional methods, often experienced reduced learner’s motivation and engagement over time, resulting in high attrition rate ^45– 48^. As such, we posit that, in an online environment where physical interactions are lacking, the TBL instructional method is better at satisfying the student’s basic needs for learning, compared to a didactic instructional method, such as lectures.

When comparing across different learning environments for TBL, we found that self-determination theory constructs, such as autonomy support, perceived competence and needs satisfaction, and perceived learning were significantly higher for in-person than in online TBL classes ( Figure 5B and 5D). Our observations support the basic tenets of SDT, which are in line with previous research that showed how TBL supports learner’s engagement and perceived learning via SDT ^13^. Similarly, a recent paper applied SDT framework to demonstrate the importance of autonomy support in enhancing K12 students’ engagement in an online learning environment during pandemic ^49^. Here, we show that online TBL classes cannot support student’s needs satisfaction to the same extent as that of in-person TBL classes.

Notably, our study showed no significant changes in the levels of engagement between online or in-person TBL classes. This suggested that the TBL instructional method could maintain student engagement across time, regardless of the learning environment. Consistent with our findings, e Silva and colleagues (2021) reported widespread acceptance of fully online TBL sessions, at comparable levels to in-person TBL sessions, across various disciplines ^50^. However, Shoair and colleagues (2023) found that students preferred in-person TBL and considered it superior to online TBL, which aligned with the themes identified by our thematic analysis ^51^.

In terms of academic performance, Anas *et al.* (2022) noted that students who attended either in-person or online TBL sessions as a supplemental activity performed better in their coursework, compared to those who did not participate in these sessions ^52^. However, when used as a replacement, there was no consensus on how student learning and performance were affected by online versus in-person TBL. Cleland *et al.* (2022) observed an increase in performances of online compared to in-person TBL in the class and end-of-year exam scores of year 2 medical students ^53^. Conversely, Babenko *et al.* (2022) reported similar learning between online and in-person TBL in a family medicine clerkship ^54^. Our results add to the growing body of literature that supports online TBL as a viable alternative when in-person classes are unavailable and highlights its potential for use in diverse educational settings.

Finally, we did not find differences in learner’s characteristics for in-person versus online TBL classes. Yet, previous studies have shown that relationships with instructors and peers in the classroom affects students’ resilience and in turn engagement, because these social relationships form the support network for the student that provides a sense of competence, relatedness and autonomy ^55– 57^. One possibility for the lack of differences in our study may be a combined effect of the use of active instructional method like TBL and the higher intrinsic motivation observed among medical students ^58^. Hence, if online TBL classes are to be implemented, instructors should still be mindful and explore ways to support these learner’s characteristics.

### What do these findings mean for online classes?

Taken together, our findings suggest that using TBL as the instructional method for an online class will help instructors to avoid the pitfalls that long-term online courses often face ^45– 48^, because online TBL is able to sustain the students’ engagement over time and at levels comparable to that of in-person TBL. This may be because the online TBL class structure retains features, such as opportunities for feedback and collaboration ^59^, that align well with the best practices in creating an autonomy-supportive classroom ^10^, which has been shown to enhance students’ self-regulation and performance ^60^. These features have been shown to promote learning accountability and responsibility during class ^61^, which in turn increases support for competence, relatedness, and autonomy ^13, 42^. Additionally, these opportunities for interaction are important for creating a sense of belonging to the learning community, which is especially important in an online learning environment, where learners may experience social isolation for a long period of time ^62, 63^. In contrast, didactic instructional methods reduce learner’s responsibility and interpersonal relations, and offers fewer opportunities for optimal challenges ^64^.

However, we noted that online TBL classes were still unable to replace in-person TBL classes, suggesting that the ‘live’ factor cannot be fully replicated in an online setting. Whilst there was no change in the levels of engagement, we found that perceived learning was higher for in-person TBL classes than online ( Figure 5D). Consistent with these findings, in our final survey question, most of the students indicated that they perceived in-person TBL to be better for their learning than online TBL ( Figure 6A). From our thematic analysis, there were two main themes of student’s perception of learning that further supported our quantitative findings: 1. Community of practice, 2. Learning space ( Figure 6B). Students valued the in-person opportunities because the socialization and team building opportunities contributed to being part of the community of practice. Importantly, in-person TBL promoted the sense of belonging because students could experience common goals in the same physical space. Similarly, recent studies showed a greater preference for in-person TBL than online TBL, with poorer ratings for teamwork interdependence in online TBL environment compared to in-person TBL ^65, 66^. Another recent study also reported similar benefits of in-person education compared to online education ^67^. Given that the sense of belonging to the community of practice is critical for professional identity formation ^68, 69^, having solely online TBL classes might affect this crucial developmental process for future physicians. Furthermore, the second identified theme of learning space also interacts with the community of practice theme. In addition, this theme suggested that the spatial identity of a learning environment influenced the learner’s perceived learning. Previous studies have shown that the design of a learning space was shown to influence engagement, motivation, professional preparation, and knowledge transfer of learners ^70– 72^. Thus, it is conceivable that when a TBL class is conducted in-person, instead of remotely, there is a significant impact on learning.

### Caveats

There are two limitations to our study. First, our institute’s safe management measures implemented in response to the pandemic precluded an in-person TBL class for the full cohort of students. Hence, the participants would attend in-person TBL on a rotational basis in a synchronous hybrid TBL format. This meant that even though they are in class, with their teammates, administrative staff and facilitating instructor, some of their classmates would be online. We have tried to mitigate the impact of such hybrid TBL arrangement by ensuring that every participant experienced a similar number of in-person TBL classes before responding to the survey. Additionally, we gave explicit instructions in the survey for participants to respond based on their experience from the in-person classes. However, we cannot rule out the possibility of unknown intermediate effects that may have an impact our findings. Second, our study data is obtained from self-reported surveys administered during the COVID-19 pandemic, when the stress due to the general uncertainty of the situation and social isolation could have an impact on students’ psychological states ^73^. Hence, our data may not be representative of the perception of online and in-person TBL classes during normal times.

## Conclusions

Taken together, our findings suggested that while online TBL was able to maintain students’ engagement, some of the basic psychological needs constructs, such as perceived competence and needs satisfaction, and perceived learning were significantly lower than in-person TBL. Similar to previous studies, our students also felt that online classes could not replace in-person classes ^74^. Hence, we suggest that if classes must be held online, instructors could consider implementing the TBL instructional method, with in-person opportunities be provided where possible ^4^. Considering the limitations of our study implementation and design, we recommend that future studies focus on obtaining objective measures of the SDT constructs, learner’s characteristics and learning gain, with a cleaner online and in-person intervention in a cross-over experimental design, to shed more light into supporting students in an online learning environment.

## Data Availability

The datasets generated and analysed during the current study are not publicly available due to university policy. According to our institutional policies, data generated from any research project carried out by NUS staff or students are the property of NUS and university staff must not share data with other parties. Permission must be sought from PI, Head of Department and NUS Research Compliance and Integrity Office. Therefore, data can be made available from the corresponding author on a reasonable request and clearance with respective parties. Figshare: ExtendedData1_Survey for online sessions.pdf https://doi.org/10.6084/m9.figshare.21982223.v1 ^25^ This project contains the following extended data: ExtendedData1_Survey for online sessions.pdf Figshare: ExtendedData2_Final survey for in person sessions.pdf https://doi.org/10.6084/m9.figshare.21982226.v1 ^24^ This project contains the following extended data: ExtendedData2_Final survey for in person sessions.pdf Data are available under the terms of the Creative Commons Attribution 4.0 International license (CC-BY 4.0).
