# Supplementary material for: A mixed methods, longitudinal study: characterizing the differences in engagement and perceived learning of medical students in online and in-person team-based learning classes
Source: MedEdPublish (2016). 2024 Jul 11;13:33. Originally published 2023 Jul 5. [Version 3] doi: 10.12688/mep.19535.3 (PMC11320038; doi:10.12688/mep.19535.3)
Supplement: Peer review history

# Reviewer report: Reviewer response for version 3

**DOI:** 10.21956/mep.21971.r38094

**Published:** 2024-09-10

**Authors:** Esin Ergonul

**Affiliations:** 1 Department of Medical Education, Dokuz Eylul Universitesi, Izmir, İzmir, Turkey

This paper provides a detailed account of how the authors adapted their school's TBL approach to an online format during the pandemic, and then moved to a hybrid model (a combination of online and face-to-face learning) once the restrictions were lifted. The authors propose that student engagement and perceived learning would be higher in face-to-face TBL sessions than in online settings. To test this hypothesis, they conducted a series of surveys measuring student engagement and perceived learning at two different points in time in both learning environments. Over the course of approximately 8-9 months, the results from the online and face-to-face environments were compared. The study provides valuable insights into the effects of hybrid and face-to-face learning on students' participation and learning experiences after the period of online education during the pandemic, and provides a comparative analysis of these different learning contexts.

 The study is generally presented in a clear and accurate manner, with adequate references to the relevant literature.

 However, additional details could be provided regarding the course content, assessment questions, and learning objectives outlined in the methodology.

 Overall, the findings seem to be well-supported by the evidence provided.

Have any limitations of the research been acknowledged?

Yes

Is the work clearly and accurately presented and does it cite the current literature?

Yes

If applicable, is the statistical analysis and its interpretation appropriate?

Yes

Are all the source data underlying the results available to ensure full reproducibility?

Yes

Is the study design appropriate and is the work technically sound?

Yes

Are the conclusions drawn adequately supported by the results?

Yes

Are sufficient details of methods and analysis provided to allow replication by others?

Yes

Reviewer Expertise:

Medical education

I confirm that I have read this submission and believe that I have an appropriate level of expertise to confirm that it is of an acceptable scientific standard.

# Reviewer report: Reviewer response for version 3

**DOI:** 10.21956/mep.21971.r38095

**Published:** 2024-09-03

**Authors:** Sarah Hyde

**Affiliations:** 1 School of Rural Medicine, Charles Sturt University, Albury, New South Wales, Australia; 2 Education, Learning, and Assessment, The Royal Australasian College of Physicians (Ringgold ID: 7796), Sydney, New South Wales, Austria

This paper provides an account of how the authors adapted their school’s TBL experience during the pandemic to an online format, and then once restrictions were lifted, to a hybrid of online and face to face. The authors have postulated that engagement and perceived learning of medical students was higher in the in-person TBL classes and used a series of surveys to measure these constructs at two time points in each learning environment, comparing results over 8-9 months.

 Although the study is well intentioned and has generated interesting ideas for adapting TBL to an online format, the study design has several limitations which make the conclusions somewhat flawed. The first concern I had was that the authors use the phrase “in-person TBL” throughout and it was not until the Methods section that I learned it was in fact more of a hybrid format than “in-person”. The authors do state that they instructed the survey respondents to think about the in-person lessons only when responding, but this is a major limitation that needs more acknowledgement.

 The potential for this paper to generate interest and impact lies in the messaging of the Introduction where it is stated that the findings can “help to identify key elements needed to support learners in a prolonged online learning environment and inform future integration of online learning into the existing medical curriculum”. The idea of learning space and separating this by spatial identity for learning and convenience and comfort level was novel and I would have liked to read more about this finding in Figure 6. The other significant point being made is that online TBL is a viable alternative when in-person classes are unavailable. More description of the set up of the online TBL is required though in order to make meaningful comparisons down the track with other online TBL research.

 I was curious to know how the results would differ if the study was repeated in the current climate where there are no restrictions. It might be reasonable to expect that the students may have felt safer completing TBL online in the pandemic, and it was also novel, whereas now it is the ‘new norm’ in some cases. In making conclusions about online versus in-person learning, the broader context matters.

**I would like to recommend the following improvements for consideration:**

  

1. Make it clearer in the abstract and introduction, and throughout the whole paper, that the second learning environment was a hybrid one – as mentioned in Figure 3. In the Discussion claims are made about the negative comparison between online and in-person when it is really about a comparison between online and hybrid environments. Some of the claims made in the Discussion and Conclusion are therefore erroneous.
2. The number of students in the sample is quite small, and although I am not a statistician I do wonder about the usefulness of parametric tests in this situation.
3. The research could have been made stronger with triangulation by comparing the student self-reports with the observations of the TBL facilitators and student performance on assessments.
4. The conclusions made, especially in the Abstract need to be more attenuated and circumspect as a result of all the above points. The claim that “ online TBL is unable to fully support the students’ needs and perceived learning” in the conclusion of the abstract is over stating the findings and is misleading when in fact at the time the study was conducted the online implementation of TBL did in fact meet their learning needs given the circumstances, and for students who require more flexibility in learning, such as mature-aged or those with a degree of physical or mental illness, online TBL might meet their learning needs to a greater extent than in-person.
5. More detail is required about the characteristics of the course at that time – for example, were there any exams in the 8 month period?
6. The paper could be strengthened by incorporating research and theory from the Community of Inquiry framework – see for example Garrison and Anderson’s work https://coi.athabascau.ca/coi-model/ The authors seem to be presenting a biased viewpoint of online learning rather than a more balanced argument – online engagement can in fact be quite high when the principles of CoI are implemented.
7. The theoretical framework presented in Figure 2 is more of a study design, consider re-labelling it as such.
8. In the Methods section – please describe how many questions the entire survey included altogether and how long it took to complete. Was it implemented online or paper-based or both?
9. Also in the Methods – I am not sure about the efficacy of combing the three subscales (autonomy, competence, relatedness) of the Basic Psychological Needs Scale into one general needs satisfaction scale. As this is the basis of the results and conclusions more information about the validity of doing this is warranted, and if no information is available about this it needs to be mentioned as a major limitation and less emphasis and confidence in the results need to be declared. Those three subscales can’t be conflated if they have already been validated in a factor analysis as separate constructs.
10. The fact that the ‘mixed’ component of study consists of a single open-ended question needs to be declared early on at the first mention of this being a mixed methods study. It is not really mixed methods in the true sense of the study design and is misleading to say so at the beginning.
11. In the Methods – describe the group composition in more detail n- was it purposefully created with a mix of gender, age, and balanced for educational background? Did the groups remain the same for the whole year or were they mixed in the hybrid situation? Did they change each term?
12. What was the matched response rate like? Did all students completed both surveys at each time point? Was there much attrition in the survey respondents between time 1 and time 2 in each setting?
13. In presenting the results of the thematic analysis, each quote needs to be identified with a participant number, as theoretically they could have all been from a single participant.
14. On page 13 of the Discission, in the third paragraph, it is stated that “the TBL instructional method could maintain student engagement across time, regardless of learning environment” – this is a key finding which is supported by the results. However, it does seem odd that engagement can be maintained despite needs satisfaction to being met, as claimed earlier. This also relates back to point 9 above and the doubtful efficacy of combining those subscales into one needs satisfaction scale and is what may be leading to these conflicting results. I suggest the authors rethink or get some expert advice on the usefulness of combining those subscales or revise back the claims and conclusions made in relation to the findings about them.

Have any limitations of the research been acknowledged?

Yes

Is the work clearly and accurately presented and does it cite the current literature?

Partly

If applicable, is the statistical analysis and its interpretation appropriate?

I cannot comment. A qualified statistician is required.

Are all the source data underlying the results available to ensure full reproducibility?

Yes

Is the study design appropriate and is the work technically sound?

Partly

Are the conclusions drawn adequately supported by the results?

No

Are sufficient details of methods and analysis provided to allow replication by others?

Partly

Reviewer Expertise:

Educational psychology, research methods, PBL, clinical reasoning, medical education, evaluation

I confirm that I have read this submission and believe that I have an appropriate level of expertise to confirm that it is of an acceptable scientific standard, however I have significant reservations, as outlined above.

# Reviewer report: Reviewer response for version 3

**DOI:** 10.21956/mep.21971.r38092

**Published:** 2024-08-12

**Authors:** Silvia Lizett Olivares Olivares

**Affiliations:** 1 Tecnologico de Monterrey, Monterrey, Nuevo Leon, Mexico

The article shows quantitative and qualitative evidence that students prefer face-to-face over online learning when applying TBL.  

 The theoretical framework for Self-Determination Theory (SDT) was adequate selection to measure and compare learning contexts. Results and conclusions are aligned with that framework. 

 However, there is more literature related to self-regulation and motivation. The authors may include other authors to complement the review.

Have any limitations of the research been acknowledged?

Yes

Is the work clearly and accurately presented and does it cite the current literature?

Yes

If applicable, is the statistical analysis and its interpretation appropriate?

I cannot comment. A qualified statistician is required.

Are all the source data underlying the results available to ensure full reproducibility?

Yes

Is the study design appropriate and is the work technically sound?

Yes

Are the conclusions drawn adequately supported by the results?

Yes

Are sufficient details of methods and analysis provided to allow replication by others?

Yes

Reviewer Expertise:

Medical education. Competences. Assessment

I confirm that I have read this submission and believe that I have an appropriate level of expertise to confirm that it is of an acceptable scientific standard.

# Reviewer report: Reviewer response for version 2

**DOI:** 10.21956/mep.21646.r36010

**Published:** 2024-02-22

**Authors:** Vinod Govind Nambiar

**Affiliations:** 1 College of Medicine and Health Sciences, National University of Science & Technology, Sohar, Oman

This is an important study about TBL as an effective means of active teaching-learning strategy, be it an online or in-person approach. 

 The authors have meticulously revised the article incorporating all the revision suggestions and the article has come out very well. This revision has given more clarity on the pre-clinical curriculum, TBL process, and the timeline of study.  Online TBL versus in-person TBL has been compared to reflect the aim of the study.

 So, there are no more comments about the article and its accepted.

Have any limitations of the research been acknowledged?

Yes

Is the work clearly and accurately presented and does it cite the current literature?

Yes

If applicable, is the statistical analysis and its interpretation appropriate?

I cannot comment. A qualified statistician is required.

Are all the source data underlying the results available to ensure full reproducibility?

No

Is the study design appropriate and is the work technically sound?

Yes

Are the conclusions drawn adequately supported by the results?

Yes

Are sufficient details of methods and analysis provided to allow replication by others?

Yes

Reviewer Expertise:

Microbiology & Immunology, Infection control, Medical education, Quality Assurance in higher education

I confirm that I have read this submission and believe that I have an appropriate level of expertise to confirm that it is of an acceptable scientific standard.

# Reviewer report: Reviewer response for version 1

**DOI:** 10.21956/mep.20929.r34741

**Published:** 2024-08-23

**Authors:** Chris Roberts

**Affiliations:** 1 Medical Education, The University of Sheffield, Sheffield, England, UK

I found this article an interesting read, and there is much of merit in this evaluative research **. **It addresses the transition to online education during the COVID-19 pandemic, focusing on the impact of different learning environments on students' motivation, engagement, and perceived learning.   The main instructional method is TBL.

 The authors use Self-Determination Theory (SDT) framework which ensures that the study's purpose, methods, and outcomes are logically connected to well established existing theory. The research questions are clearly stated, focusing on the impact of different learning environments on students' motivation, engagement, and perceived learning. The use of a mixed methods approach is a strength, as it combines quantitative and qualitative data to provide a comprehensive view of the research questions.  The results were clearly presented. The discussion summarised the key findings and reported how this research compared/added to the existing literature. There is a discussion of the implications for educators and future research. The limitations of the study are discussed.

 Potential areas for strengthening include.

1. Further develop the intro stating the problem in the research context and identifying the gap in the international research literature, and emphasising how this study addresses those challenges?
2. More clarity over the quasi-experimental design comparing online and in-person TBL groups. It can be a little difficult to separate the impact of the local contextual factors from the research design, and whether students could realistically only take into consideration their experiences for the in-person sessions when completing the surveys. Can the authors claim an effect rather than an association?
3. Provide more information about the coding process steps taken to ensure the trustworthiness and credibility of qualitative findings from the theoretical lens of SDT.
4. Some more info on the validity of the chosen research instruments and justification of the use of many scales in relation to the specific hypothesis that was stated and a small population of students.   
5. More emphasis on the key findings of this research in the opening discussion section in the light of the SDT and an integration of the qual and quant findings.
6. The article briefly mentioned some caveats, but it could elaborate on the methodological challenges of the study, including the sample size and potential confounding variables. Also, to take the opportunity to mention the strengths of the study.
7. For those schools who for various reasons are still using on line TBL and face to face is not fully possible are there implications for them?

Have any limitations of the research been acknowledged?

Yes

Is the work clearly and accurately presented and does it cite the current literature?

Yes

If applicable, is the statistical analysis and its interpretation appropriate?

Yes

Are all the source data underlying the results available to ensure full reproducibility?

Yes

Is the study design appropriate and is the work technically sound?

Yes

Are the conclusions drawn adequately supported by the results?

Partly

Are sufficient details of methods and analysis provided to allow replication by others?

Partly

Reviewer Expertise:

Assessment, instructional methods, professionalism, quant and qual methodologies

I confirm that I have read this submission and believe that I have an appropriate level of expertise to confirm that it is of an acceptable scientific standard, however I have significant reservations, as outlined above.

# Reviewer report: Reviewer response for version 1

**DOI:** 10.21956/mep.20929.r34740

**Published:** 2024-05-25

**Authors:** Sarah Leupen

**Affiliations:** 1 University of Maryland Baltimore County, Baltimore, Maryland, USA

In this paper the authors compared in-person and online experiences of students using a pedagogy that takes some aspects, specifically the readiness assurance process, from the Team-Based Learning pedagogical strategy. Students experienced the pedagogy online, and then experienced it in person (with some of their colleagues still online), and some aspects of their experience and performance improved when they were in person.

 I have two major criticisms, and more minor comments.

 Major issues:

 1. This seems to be a modified form of TBL with no application questions. Most TBL practitioners would argue that the application questions are the heart of TBL, and the other stuff (RAT, etc.) simply prepares them for the application questions. The RAT itself can act as a motivation to prepare for class, so it certainly has value, but this is not TBL as described.

 2. The in-person students experienced TBL more positively. But they were also just older and had more TBL, so we don’t know if it was the format, their experience in medical school, age, experience with TBL, etc. that caused the difference. To find out the answer to their question, they should simply give the survey to current first-year students who are presumably learning in person. Then you are comparing apples to apples: students in their first semester of medical school and first semester using TBL.

 Related to the above, comments on the Discussion and Conclusions are as follows:

 Discussion: the conclusion of the first paragraph (“In this study…”) is warranted.

 The conclusion of the second paragraph (“When comparing…”) is not, since the students experiencing the in-person class are the same students later in the class/curriculum, as noted above; there is no real control, and it is likely that they were simply delighted to be back in person after COVID. This aspect could be corrected by a crossover design in which a new cohort of students have in-person followed by online (if considered ethical), or simply by comparing students in their first semester now, in person, to the previous students who experienced their first semester online, in this study. (Later, the authors recommend this themselves—great!) Also, I don’t think it’s appropriate to compare to other TBL studies, since this is only RATs, not TBL itself, which is centered on application exercises, with the RATs functioning only to prepare students for those applications. This could be corrected by either labeling this pedagogy as one that has aspects of TBL practice in it, or otherwise described according to what happened without reference to TBL, or by clarifying, if true, that there *were* actually application exercises that took up the bulk of class time, whether in-person or online.

 It is also possible that the online TBL was not carried out as effectively as it could have been, which is perfectly understandable since we were all scrambling to do the best we could, but could mean that TBL *could* be carried out effectively online, but might not have been here. Certainly many TBL practitioners believe that TBL can be effective in fully online environments.

 I agree with the authors that the relatively high level of engagement in the online class is an indication that didactic methods are inferior to active learning in this way. That is already well-established, but additional confirmation is always useful. So, the first paragraph of “What do these findings mean” is supported (except maybe the TBL label).

 The findings described in the second paragraph of that section provide reasons to explore the differences in online vs. in-person TBL (or whatever this pedagogy is exactly) in a controlled way. I would look forward to reading that study.

 We know that perceived learning and actual learning are often different, especially in an active learning context (see DesLauriers et al. 2017 for example), so we don’t know if those are related here, but the authors do use careful language (“it is conceivable that”). If the authors did a more controlled study of this question, they could look at results of validated exam questions, or ideally a pre-post test, to see if actual learning did differ.

 Caveats: As noted, I think the major caveat is the uncontrolled nature of the study as described above in major issue #2.

 Conclusions: Not bad! The authors do not recommend that TBL be used in person instead of online, which indeed we can’t tell from this, but that a cross-over study be used to gather more data, which I completely agree with. They also recommend that online instructors consider TBL, which, certainly compared to a didactic method, is indeed likely to be more effective in the measures described here.

 Minor comments:

 1. The introduction needs to be restructured. You have to say what TBL is, how it works, and why you think it should be better on these scales based on self-determination theory. THEN you can cite that the paper showing that it is better. But, considering this does not seem to be ‘real’ TBL, maybe it is better to talk more generally about the pedagogy, so this criticism may depend on how the authors feel about describing this as something other than TBL.

 2. [very minor] In the Results: I would think the Cronbach’s alpha levels would not rise to the level of appearing in a table in the results, but that’s for the editor to decide. Otherwise, no major issues with the results reporting. I like the thematic analysis and Fig 6B.

 3. I didn’t understand the sentence: “a handful of students preferred online TBL as a result of dysfunctional team dynamics presented over the initial phase of prolonged online learning environment.” I’m good up to “dynamics” but if they preferred online, wouldn’t it be because the in-person learning environment was perceived as poor?

 4. Underlying data sets: I don’t care that the underlying data sets are inaccessible. We have to trust authors of papers to some degree. But I understand this is the policy of this journal, for authors to provide them.

 5. Have someone, perhaps a copyeditor, check the paper to add missing articles, especially “the,” in the appropriate places, and fix all the incorrect apostrophes. Also I think this is a writing mistake: “The increasing number of reports on the lessons learnt and efforts in the online transition ^1, 5^, particularly in terms of learners’ perception of the online learning environment ^3^, had enabled the swift implementation of online learning.” The reports came after the implementation, not at all enabling them.

Have any limitations of the research been acknowledged?

Partly

Is the work clearly and accurately presented and does it cite the current literature?

Partly

If applicable, is the statistical analysis and its interpretation appropriate?

I cannot comment. A qualified statistician is required.

Are all the source data underlying the results available to ensure full reproducibility?

No

Is the study design appropriate and is the work technically sound?

Partly

Are the conclusions drawn adequately supported by the results?

Partly

Are sufficient details of methods and analysis provided to allow replication by others?

Yes

Reviewer Expertise:

team-based learning and other evidence-based pedagogies that use teams or groups; active learning broadly to some degree

I confirm that I have read this submission and believe that I have an appropriate level of expertise to state that I do not consider it to be of an acceptable scientific standard, for reasons outlined above.

## References

[1] : Measuring actual learning versus feeling of learning in response to being actively engaged in the classroom. *Proc Natl Acad Sci U S A* .2019;116(39) : 10.1073/pnas.1821936116 19251-19257 10.1073/pnas.1821936116 31484770 PMC6765278

# Response

**Published:** 2024-07-05

**Authors:** Irene Lee

**Affiliations:** Duke-NUS Medical School, Singapore

We thank Reviewer 3 for their comments. We have noted that the reviewer was reviewing the first version of our paper. As such, some of the comments made, especially for the major concern regarding the form of TBL, have been addressed in our second version. However, we have kept Reviewer 3’s comments in mind as we revised the manuscript for version 3. We provide a point by point response highlighted in blue below. We thank Reviewer 3 again for the thoughtful review and input to improve the paper.

 I have two major criticisms, and more minor comments.

 1 ^st^ Major concern:

 - This seems to be a modified form of TBL with no application questions. - Also, I don’t think it’s appropriate to compare to other TBL studies, since this is only RATs, not TBL itself, which is centered on application exercises, with the RATs functioning only to prepare students for those applications.

 Response: We thank Reviewer 3 for pointing this out. We had missed out on representing the application section in Version 1, which was also pointed out by other reviewers. In Version 2, and now 3, our revised Figure 2 now includes the “Knowledge Application” section. We implemented the full TBL experience throughout the whole study period. A minor note, with reviewer 3 comment about introduction, this Figure is now Figure 1.

  2 ^nd^ Major concern:

 - The in-person students experienced TBL more positively. But they were also just older and had more TBL, so we don’t know if it was the format, their experience in medical school, age, experience with TBL, etc. that caused the difference. To find out the answer to their question, they should simply give the survey to current first-year students who are presumably learning in person. Then you are comparing apples to apples: students in their first semester of medical school and first semester using TBL. - The conclusion of the second paragraph (“When comparing…”) is not, since the students experiencing the in-person class are the same students later in the class/curriculum, as noted above; there is no real control, and it is likely that they were simply delighted to be back in person after COVID.

- Response: We appreciate the reviewer's insightful point regarding the influence of various factors (age, experience with TBl, etc.) on students' perceptions of TBL. This concern is precisely why we explored various characteristics, SDT framework, engagement and perceived learning, over an extended period to account for possible changes due to the time effect. Circling back to our study aim, to accurately determine these perceptions within different learning environments, we characterized differences in learner characteristics (such as resilience, curiosity, growth mindset, use of cognitive strategies, and self-regulation), basic psychological needs satisfaction (autonomy, competence, and relatedness), situational motivation (intrinsic, identified, external, and amotivation), student engagement, and perceived learning between the two TBL learning environments, online and in-person, over an extended period of time. To account for the potential effect of having more TBL sessions, we administered two surveys per learning environment. The first survey was conducted after 20 TBL sessions (early timepoint), and the second one was conducted 50 days later (late timepoint) for each learning environment. We did not detect any changes between the early and late timepoints within each learning environment. This stability in measures is shown in Table 2 and discussed in our conclusion: “In this study, we showed that the learner’s characteristics and self-determination theory constructs did not change across time in each learning environment (Table 2).” This suggests our measures were stable and unaffected by the number of TBL sessions, a point also supported by Reviewer 3.
-  On the reviewer’s point about “simply delighted coming out of COVID,” we appreciate that this is a possible phenomenon given the social isolation and other psychosocial factors that students underwent, as shared in our discussion. However, if the observed positive effects were due to this factor alone, constructs that measure the emotional perspective on perceiving positivity, delight, and joy would likely show a significant increase in self-reported ratings. We would then expect significant differences in the measures for the Situational Motivation Scale (such as intrinsic motivation), emotional engagement, and curiosity for joyous exploration. Yet, we only observed a difference for perceived learning, with in-person TBL scoring higher than in online TBL, while situational motivations, curiosity, and engagement showed no significant differences (Table 3 and Figure 3). Our reliability statistics results also added confidence to the constructs we measured (Table 1). Consistent with this finding, we did not identify any themes from students reflecting on "simply delightedness" to come out of COVID as a reason for their preferred learning environment being in-person. Therefore, our findings suggest that the positive perception of in-person TBL is not solely due to the relief of coming out of COVID, but is likely influenced by other factors inherent to the in-person TBL experience.  
- The reviewer suggested that the in-person students experience TBL more positively because they were older and had more TBL. Hence, to validate our findings, we should administer the survey the current first-year students for an “apple-to-apple comparison”.
- We thank the reviewer for the suggestion of using a cross-cohort experimental design to reduce the effect of time and experience as possible confounding factors. However, we would first like to point out that if the effect of time and experience were factors that could cause students to experience in-person TBL more positively, then we should expect to see a difference between the first time-point and second time-point surveys that were administered for the in-person TBL, after all, they would have more in-person TBL experience by the time they had to fill out the second in-person survey. However, no such differences were observed (excerpt from Table 2, with measured constructs with observed differences shown below).

1. **Measured Constructs** **In-Person 1** **(Day 75)** **In-Person 2** **(Day 125)**
2. **Mean** **SEM** **Mean** **SEM** **Autonomy** 5.32 0.11 5.28 0.12
3. **Needs Satisfaction** 3.76 0.07 3.79 0.07
4. **Perceived Competence** 4.61 0.09 4.67 0.10
5. **Perceived Learning** 3.58 0.09 3.59 0.07

- Second, it can be seen from figure 3 that the participants completed all 4 surveys in their first year of medical school.
- Summary of survey timeline: 1st online survey: 3rd month 2nd online survey: 5th month 1st in-person survey: 8th month 2nd in-person survey: 10th month If the differences observed between online and in-person TBL surveys are primarily attributable to increased experience over time, we would expect to see a larger change between the 1st online survey (3rd month) and the 1st in-person survey (8th month) compared to the change between the 2nd online survey (5th month) and the 1st in-person survey (8th month). This is because the former comparison spans a longer period (5 months) than the latter (3 months). However, we did not observe such differences (Table 2). Third, comparing across cohorts may not lead to an “apple-to-apple comparison”, because the absolute values measured from surveys could reflect different baseline levels across cohorts. These differences may arise from varying prior experiences, backgrounds, and contexts, which are challenging to account for in the current study. Such variability could confound the results, making it difficult to attribute differences solely to the learning environment, thus presenting a threat to internal validity (Shadish et al., 2002). Yet, we acknowledge that our experimental design has its weaknesses. As noted in our discussion, future studies could involve a pure cross-over experimental design to shed more light on supporting students in purely online and in-person learning environments. We also recognize that there are ways to account for baseline differences in cohort comparison, such as using propensity score matching and pretest-posttest designs. If we had administered a baseline survey, we could have been able to account for these baseline differences. Unfortunately, due to the timing and constantly changing situation during the COVID pandemic, we were unable to administer the baseline survey. Hence, we opted for within-cohort comparisons to control for these differences. Using the repeated measures ANOVA across an extended period allowed us to elucidate any changes caused by the long-term immersion in either of the learning environments, within the same cohort. Our study design provided valid findings based on our reliability statistics, and we observed specific differences in measurements, as opposed to generic changes, across different learning environments. While we appreciate the reviewer’s suggest of adopting a cross-cohort design, the additional few months needed to include the upcoming student cohort may not significantly enhance the validity of our findings at this stage.

Minor concerns:

 1.  If the authors did a more controlled study of this question, they could look at results of validated exam questions, or ideally a pre-post test, to see if actual learning did differ.

 Response: We thank the reviewer for the suggestion and will keep it in mind for future studies. For this study, all our TBL assessments were formative. As such, we focused on characterising the student’s perceptions of online versus in-person TBL, rather than actual learning.

 2. The introduction needs to be restructured. You have to say what TBL is, how it works, and why you think it should be better on these scales based on self-determination theory. THEN you can cite that the paper showing that it is better.

 Response: We thank the reviewer on the feedback of the introduction. We have moved the explanation of what TBL is from the methods to the introduction. For the rest of the introduction, while we understand reviewer’s suggestion on restructuring the introduction, the current structure effectively conveys the necessary information and builds a logical argument for the study, as we start upfront with the problem/unknown, then proposed the framework that we used to characterise the  unknown, specifying the scope and questions that we aim to answer.

 3. I didn’t understand the sentence: “a handful of students preferred online TBL as a result of dysfunctional team dynamics presented over the initial phase of prolonged online learning environment.” I’m good up to “dynamics” but if they preferred online, wouldn’t it be because the in-person learning environment was perceived as poor?

   Response: Referring to this specific section, we have revised it to provide better clarity to our point. Please see the highlighted portion for the revised text. Based on the thematic analysis, the first theme identified is that interpersonal interaction and team dynamics influence students' preference for a learning environment. For students who preferred online learning, their comments indicate that inherent tension in team dynamics shaped their preference. This insight goes beyond simply perceiving the in-person environment as poor and highlights the complexity of team interactions in different settings. Original Revised While most students who supported in-person TBL due to the abundance of interpersonal interaction with peers, a handful of students preferred online TBL as a result of dysfunctional team dynamics presented over the initial phase of prolonged online learning environment. For examples, these two students perceived being disconnected from team members and not being engaged in an in-person TBL, suggestive of inherent challenges with team cohesion and dynamics.     “Don't really feel connected to teammates… Better to focus at home instead” “While discussing things over zoom, everyone is on the same page whereas in school, discussions will leave some members out”.   While Most students who supported in-person TBL did so  due to the abundance of interpersonal interaction with peers. However, a small subset of students expressed a preference for online TBL, citing challenges with in-person team dynamics. This preference may be attributed to their extended exposure in the online learning environment, potentially leading to difficulties in adjusting to face-to-face discussions. For examples, two students perceived being disconnected from team members and less engaged when in-person.  

 4. Have someone, perhaps a copyeditor, check the paper to add missing articles, especially “the,” in the appropriate places, and fix all the incorrect apostrophes. Also I think this is a writing mistake: “The increasing number of reports on the lessons learnt and efforts in the online transition ^1^ ^,^ ^5^ , particularly in terms of learners’ perception of the online learning environment ^3^ , had enabled the swift implementation of online learning.” The reports came after the implementation, not at all enabling them.

 Response: We have gone through the paper to correct the grammatical errors. Additionally, we have rephrased the highlighted statement to as follows: “Due to the looming threat of infectious variants and the rapidly changing pandemic situation, many educational institutions implemented either online learning or a hybrid arrangement of blended in-person and online learning for a prolonged period of time ^4^ . This led to many initial reports on the lessons learnt and efforts in the online transition ^1^^, ^^5^ , particularly in terms of learners’ perception of the online learning environment ^3^. However, it remains unclear whether being in an online learning environment over an extended period changes a learner’s motivation and in turn, the level of engagement and the perception of learning.”    

 References: Shadish, W. R., Cook, T. D., & Campbell, D. T. (2002). *Experimental and quasi-experimental designs for generalized causal inference*. Houghton Mifflin.

# Reviewer report: Reviewer response for version 1

**DOI:** 10.21956/mep.20929.r34743

**Published:** 2023-10-17

**Authors:** Vinod Govind Nambiar

**Affiliations:** 1 College of Medicine and Health Sciences, National University of Science & Technology, Sohar, Oman

1. This research on TBL is noteworthy as TBL is gaining importance as a resource-efficient, student-centered active learning and teaching strategy.

 2. The authors have done good work on TBL using various validated survey instruments.

 3. The paper is well written with an appropriate analysis of the findings.

 4. However, some points in the paper which can be improved upon:

-  In the Methods section, the authors can give in few words more clarity on the 1 ^st^ year pre-clinical curriculum – duration, number of subjects/courses taught, total number of TBL classes taken semester-wise, number of assessments done, etc.(page 4)
- ‘In-person and online TBL methods’ – there is no mention of the “Knowledge Application step” component of the TBL process. Whether ‘instructor-facilitated discussion’ and ‘Application step’ is the same?(page 4)
- Experimental design – A flowchart on study cohorts, teams, timelines, and surveys administered can give more clarity.
- Figure 4 – Each team included only study participants or was there a mix of study participants and non-participants.
- Results – Whether instructors were present in the same classroom to facilitate discussions?  However, it’s mentioned that – ‘such interaction with faculty remains limited as the faculty did not co-share the same physical learning environment with the students, given the prevailing social distancing measures on teaching venue. Thus, this was highlighted in students who found no difference between the two learning environments. (page 9) But on page 4, it is mentioned that ‘The administrative staff and facilitating instructors would be present in the classroom’.
- Discussion – Needs to be more focused on the aims of the study: ‘to characterize differences in the two learning environments of TBL -online vs. In-person’.
  However, a comparison between TBL and didactic lectures is made in some areas (page 11), but student characteristics during didactic lectures were not studied in this research.
  Was TBL activity graded/assessed at the end of the semester or at the end of 1 ^st^ year? Whether this will impact student participation in the study and survey?  
- The authors have clearly noted the limitations of the study and the conclusions of the study.

Have any limitations of the research been acknowledged?

Yes

Is the work clearly and accurately presented and does it cite the current literature?

Yes

If applicable, is the statistical analysis and its interpretation appropriate?

I cannot comment. A qualified statistician is required.

Are all the source data underlying the results available to ensure full reproducibility?

No

Is the study design appropriate and is the work technically sound?

Yes

Are the conclusions drawn adequately supported by the results?

Yes

Are sufficient details of methods and analysis provided to allow replication by others?

Yes

Reviewer Expertise:

Microbiology & Immunology, Infection control, Medical education, Quality Assurance in higher education

I confirm that I have read this submission and believe that I have an appropriate level of expertise to confirm that it is of an acceptable scientific standard, however I have significant reservations, as outlined above.

# Response

**Published:** 2024-02-06

**Authors:** Irene Lee

**Affiliations:** Duke-NUS Medical School, Singapore

We thank the reviewer for the positive comments and constructive suggestions. We have addressed the reviewer's comments and suggestions point-by-point as outlined below:

-  In the Methods section, the authors can give in few words more clarity on the 1 ^st^ year pre-clinical curriculum – duration, number of subjects/courses taught, total number of TBL classes taken semester-wise, number of assessments done, etc.(page 4)

Response: We thank the reviewer for highlighting this. For clarity, we have expanded the description on our preclinical curriculum in the Methods section.

  

- ‘In-person and online TBL methods’ – there is no mention of the “Knowledge Application step” component of the TBL process. Whether ‘instructor-facilitated discussion’ and ‘Application step’ is the same?(page 4)

Response: We thank the reviewer for pointing this out and have revised Figure 2 to include “Knowledge Application”, which is the knowledge application step of the TBL process.  

- Experimental design – A flowchart on study cohorts, teams, timelines, and surveys administered can give more clarity.

Response: We have revised Figure 3 to provide spatial separation between the different sets of information, such that the timeline of the study, and when the surveys are administered relative to the background of the academic semester and reopening of Singapore during the COVID pandemic can be appreciated. Additionally, as mentioned in our methods, this is a quasi-design study, with only 1 study cohort, and the teams in this cohort were illustrated in Figure 4.

  

- Figure 4 – Each team included only study participants or was there a mix of study participants and non-participants.

Response: The teams were formed prior to the recruitment of participants for this study, therefore there was a mix of study participants and non-participants in each team. This study was designed to have minimal disruption on the running of the classes and the students’ learning experiences, with the surveys being the main tool used to gather data, which were administered outside of class time.

  

- Results – Whether instructors were present in the same classroom to facilitate discussions?  However, it’s mentioned that – ‘such interaction with faculty remains limited as the faculty did not co-share the same physical learning environment with the students, given the prevailing social distancing measures on teaching venue. Thus, this was highlighted in students who found no difference between the two learning environments. (page 9) But on page 4, it is mentioned that ‘The administrative staff and facilitating instructors would be present in the classroom’.

Response: As the reviewer has rightly noted, administrative staff and facilitating instructors were indeed present in the classroom. These groups of people are different from the faculty, who take on the role of content experts in each TBL class. As most of our faculty are clinical faculty, during COVID, due to the cohorting and other safe-distancing measures, the clinical faculty could not come to the campus, hence, they were not present in the same classroom as the students. This might be the reason why some students perceived no difference between the two learning environments.

- Discussion – Needs to be more focused on the aims of the study: ‘to characterize differences in the two learning environments of TBL -online vs. In-person’.
  However, a comparison between TBL and didactic lectures is made in some areas (page 11), but student characteristics during didactic lectures were not studied in this research.

Response: We thank the reviewer for this suggestion and have revised the discussion to redirect the focus to online and in-person TBL. We have also added latest references to discuss such differences.   

- Was TBL activity graded/assessed at the end of the semester or at the end of 1 ^st^ year? Whether this will impact student participation in the study and survey?  

          Response: TBL assessments were all formative. As such, we do not expect any impact of grades on student participation in the study and survey. All students, whether study participants or not, had access to the same learning resources throughout the study.

# Reviewer report: Reviewer response for version 1

**DOI:** 10.21956/mep.20929.r34200

**Published:** 2023-08-24

**Authors:** Alam Sher Malik

**Affiliations:** 1 Management and Science University, Shah Alam, Selangor, Malaysia

- This paper addresses a very important aspect in relation to teaching/learning methods and approaches.
- Authors have worked meticulously to get the information through the surveys and analysis thereafter.
- It is amazing to note that there was no drop-out among the participants during the study.
- Sections on “Introduction” and “Results” have been very well written.

However, there are following concerns:

- The TBL process seems to be truncated as shown in figure 2. There is no mention of “Team Application” step in the process. Although in the text it is mentioned as follows:
   
  - “Students would be put in breakout rooms when they are doing the team readiness assurance tests and working through the clarification questions”. (Page 4).
- It is not clear, if it is the same cohort of students in both for online and in-person TBL classes.
- In figure 3 two different dates are mentioned in the diagram and text below it i.e. 27 ^th^ July and 20 ^th^ August 2020.
- First survey was conducted after twenty TBL classes. How many sessions were held before conducting the second survey?
- In discussion section a comparison has been drawn between TBL and other methods of teaching e.g., didactic lectures – which is not the states focus of this paper.

Have any limitations of the research been acknowledged?

Yes

Is the work clearly and accurately presented and does it cite the current literature?

Yes

If applicable, is the statistical analysis and its interpretation appropriate?

I cannot comment. A qualified statistician is required.

Are all the source data underlying the results available to ensure full reproducibility?

No

Is the study design appropriate and is the work technically sound?

Yes

Are the conclusions drawn adequately supported by the results?

Partly

Are sufficient details of methods and analysis provided to allow replication by others?

Yes

Reviewer Expertise:

Medical education; Paediatric Medicine

I confirm that I have read this submission and believe that I have an appropriate level of expertise to confirm that it is of an acceptable scientific standard, however I have significant reservations, as outlined above.

# Response

**Published:** 2024-02-06

**Authors:** Irene Lee

**Affiliations:** Duke-NUS Medical School, Singapore

We thank the reviewer for the positive comments and constructive suggestions. We have addressed the reviewer’s comments and suggestions point-by-point as outlined below in blue. Response to Reviewer 1

- The TBL process seems to be truncated as shown in figure 2. There is no mention of “Team Application” step in the process.

Response: We thank the reviewer for pointing this out and have revised Figure 2 to include “Knowledge Application”.

- It is not clear, if it is the same cohort of students in both for online and in-person TBL classes.

Response: It is the same cohort of students, as can be seen from Figure 3, where the study took place between 2020 to 2021, spanning 2 academic semesters within the same Academic Year 2020/2021. Also, in the text “During this study period, the participants started their Year 1, semester 1, in the online TBL environment, due to the heightened social distancing measures during the COVID-19 pandemic. In semester 2, as the infection clusters came under control, social distancing measures were relaxed sufficiently for half the cohort to attend lessons in-person, with the other half attending synchronously on Zoom.”

- In figure 3 two different dates are mentioned in the diagram and text below it i.e. 27 ^th^ July and 20 ^th^ August 2020.

Response: The revised Figure 3 now clearly delineates the timeline of Singapore's reopening during the COVID-19 pandemic, the University's COVID-19 measures, the academic semester at Duke-NUS, and our study timeline. Specifically, it shows that Singapore began its reopening phase on 27th July 2020, as indicated by the grey text beneath this date, allowing small group gatherings. Furthermore, the figure highlights that the first semester at Duke-NUS commenced in August 2020. This spatial separation in the figure should aid in distinguishing these key timelines.

  

- First survey was conducted after twenty TBL classes. How many sessions were held before conducting the second survey?

Response: We conducted the first survey following twenty Team-Based Learning (TBL) sessions. This timing was chosen to ensure participants had ample opportunity to familiarize themselves with their teammates and fully engage with the TBL method. The second survey was administered 50 days later to assess the impact of being immersed in a learning environment for a prolonged period. In the second semester, despite a change in the learning environment, we maintained the same schedule for the first survey and the same 50-day interval before conducting the second survey.

- In discussion section a comparison has been drawn between TBL and other methods of teaching e.g., didactic lectures – which is not the states focus of this paper.

Response: We thank the reviewer for this suggestion and have revised the discussion to redirect the focus to online and in-person TBL.
